# Translation of the circular RNA circβ-catenin promotes liver cancer cell growth through activation of the Wnt pathway

**DOI:** 10.1186/s13059-019-1685-4

**Published:** 2019-04-26

**Authors:** Wei-Cheng Liang, Cheuk-Wa Wong, Pu-Ping Liang, Mai Shi, Ye Cao, Shi-Tao Rao, Stephen Kwok-Wing Tsui, Mary Miu-Yee Waye, Qi Zhang, Wei-Ming Fu, Jin-Fang Zhang

**Affiliations:** 1 Vaccine Research Institute, The Third Affiliated Hospital of Sun Yat-sen University, Sun Yat-sen University, Guangzhou, People’s Republic of China; 2 School of Biomedical Sciences, The Chinese University of Hong Kong, Shatin, New Territories, Hong Kong, People’s Republic of China; 3 School of Life Sciences, Sun Yat-sen University, Guangzhou, People’s Republic of China; 4 The Nethersole School of Nursing, The Chinese University of Hong Kong, Shatin, New Territories, Hong Kong, People’s Republic of China; 5 School of Pharmaceutical Sciences, Southern Medical University, Guangzhou, 511458 People’s Republic of China; 6 Guangdong Provincial Key Laboratory of New Drug Screening, School of Pharmaceutical Sciences, Southern Medical University, Guangzhou, 510515 People’s Republic of China; 7 Key Laboratory of Orthopaedics and Traumatology, The First Affiliated Hospital of Guangzhou University of Chinese Medicine, The First Clinical Medical College, Guangzhou University of Chinese Medicine, Guangzhou, People’s Republic of China; 8 Lingnan Medical Research Center, Guangzhou University of Chinese Medicine, Guangzhou, 510405 People’s Republic of China

**Keywords:** Circular RNA, Non-coding RNA, Coding capacity, Wnt pathway, Cell growth

## Abstract

**Background:**

Circular RNAs are a class of regulatory RNA transcripts, which are ubiquitously expressed in eukaryotes. In the current study, we evaluate the function of a novel circRNA derived from the β-catenin gene locus, circβ-catenin.

**Results:**

Circβ-catenin is predominantly localized in the cytoplasm and displays resistance to RNase-R treatment. We find that circβ-catenin is highly expressed in liver cancer tissues when compared to adjacent normal tissues. Silencing of circβ-catenin significantly suppresses malignant phenotypes in vitro and in vivo, and knockdown of this circRNA reduces the protein level of β-catenin without affecting its mRNA level. We show that circβ-catenin affects a wide spectrum of Wnt pathway-related genes, and furthermore, circβ-catenin produces a novel 370-amino acid β-catenin isoform that uses the start codon as the linear β-catenin mRNA transcript and translation is terminated at a new stop codon created by circularization. We find that this novel isoform can stabilize full-length β-catenin by antagonizing GSK3β-induced β-catenin phosphorylation and degradation, leading to activation of the Wnt pathway.

**Conclusions:**

Our findings illustrate a non-canonical function of circRNA in modulating liver cancer cell growth through the Wnt pathway, which can provide novel mechanistic insights into the underlying mechanisms of hepatocellular carcinoma.

**Electronic supplementary material:**

The online version of this article (10.1186/s13059-019-1685-4) contains supplementary material, which is available to authorized users.

## Background

As a global health problem, liver cancer is a devastating disease with high mortality rate. The known etiologies for liver cancer include chronic virus infection, cirrhosis, long-term alcohol abuse, hepatic steatosis, and aflatoxin exposure [1]. The high mortality rate of liver cancer is attributed to lack of effective diagnostic tools as well as efficient therapeutic approaches, especially for the cancer patients in advanced stages [2]. Despite extensive exploration, the underlying molecular mechanisms during liver cancer progression remain obscure.

In the past decades, numerous signaling pathways have been identified to get involved in hepatocarcinogenesis, including Notch pathway, PI3K/Akt pathway, and Wnt/β-catenin pathway [3–5]. As a highly conserved pathway, Wnt/β-catenin pathway extensively participates in a variety of pathological events, such as tumor growth, escape from programmed cell death, cell fate determination, and maintenance of self-renewal capacity [6]. In the absence of Wnt ligands, cytosolic β-catenin is phosphorylated by casein kinase Iα (CKIα) at Ser45 sites, which subsequently primes β-catenin phosphorylation at three residues (Ser33/Ser37/Thr41) by glycogen synthase kinase 3β (GSK3β). Upon phosphorylation, cytosolic β-catenin is sequestered by the destruction complex consisting of APC, GSK3β, and Axin, followed by proteasome degradation. However, when the Wnt ligands bind to frizzled receptors, the cytosolic β-catenin is released from the destruction complex and translocates from cytoplasm towards the nucleus. The nuclear β-catenin interacts with TCF/LEF complex and subsequently initiates the transcription of target genes [3, 7]. Previous investigations indicate that aberrant activation of β-catenin occurs in 40~ 70% of liver cancer patients and hyperactivation of β-catenin frequently correlates with poor prognosis [8, 9]. Furthermore, based on high-throughput platform, it was found that several β-catenin downstream target genes also displayed high expression in chronic liver diseases as well as liver cancer. For instance, amplification of cyclin D1 and c-myc in liver cancer is frequently associated with enhanced nuclear β-catenin accumulation along with unfavorable clinical outcome [10, 11]. Despite that the underlying molecular mechanisms of Wnt/β-catenin pathway have been extensively explored, the reason why it is hyper-activated in liver cancer tissues is still poorly understood.

The regulatory RNA world includes a wide spectrum of RNA molecules such as microRNA, Piwi-interacting RNA, small interfering RNA, and long non-coding RNA. With the recent advances in high-throughput sequencing techniques and bioinformatics tools, numerous circular RNAs (circRNAs) have been identified in various human tissues and cell lines [12, 13]. The circRNAs are produced by circularization of specific exons and thus harbor covalently closed loop structure, which means that they do not have 5′ to 3′ polarity and Poly(A) tails [14, 15]. It is found that the majority of circRNAs originates from protein-coding genes [16, 17]. Despite that a large number of circRNAs have been identified so far, the biological functions of only a few circRNAs have been illustrated. It was reported that circRNAs exerted their function by serving as miRNA sponges or modulating the gene transcription of their cognate mRNAs [14, 18–20]. However, the function of circRNAs is still largely unexplored. Recent studies suggest that circRNAs are translatable and they can produce previously unknown protein isoforms [21–25]. Nonetheless, the experimental data which can support this hypothesis are scarce.

In this study, we analyzed the RNA sequencing data from an online database and identified a circRNA derived from the β-catenin gene, which was named “circβ-catenin.” Functional investigations showed that knockdown of circβ-catenin suppressed in vivo and in vitro cancer cell growth. Subsequent studies displayed that circβ-catenin encoded a novel 370-amino acid β-catenin isoform. This isoform protected β-catenin from GSK3β-mediated degradation and thus potentiated the activation of Wnt/β-catenin pathway.

## Results

### Bioinformatics analysis of Wnt pathway-related circRNAs in human cell lines

**Fig. 1:**
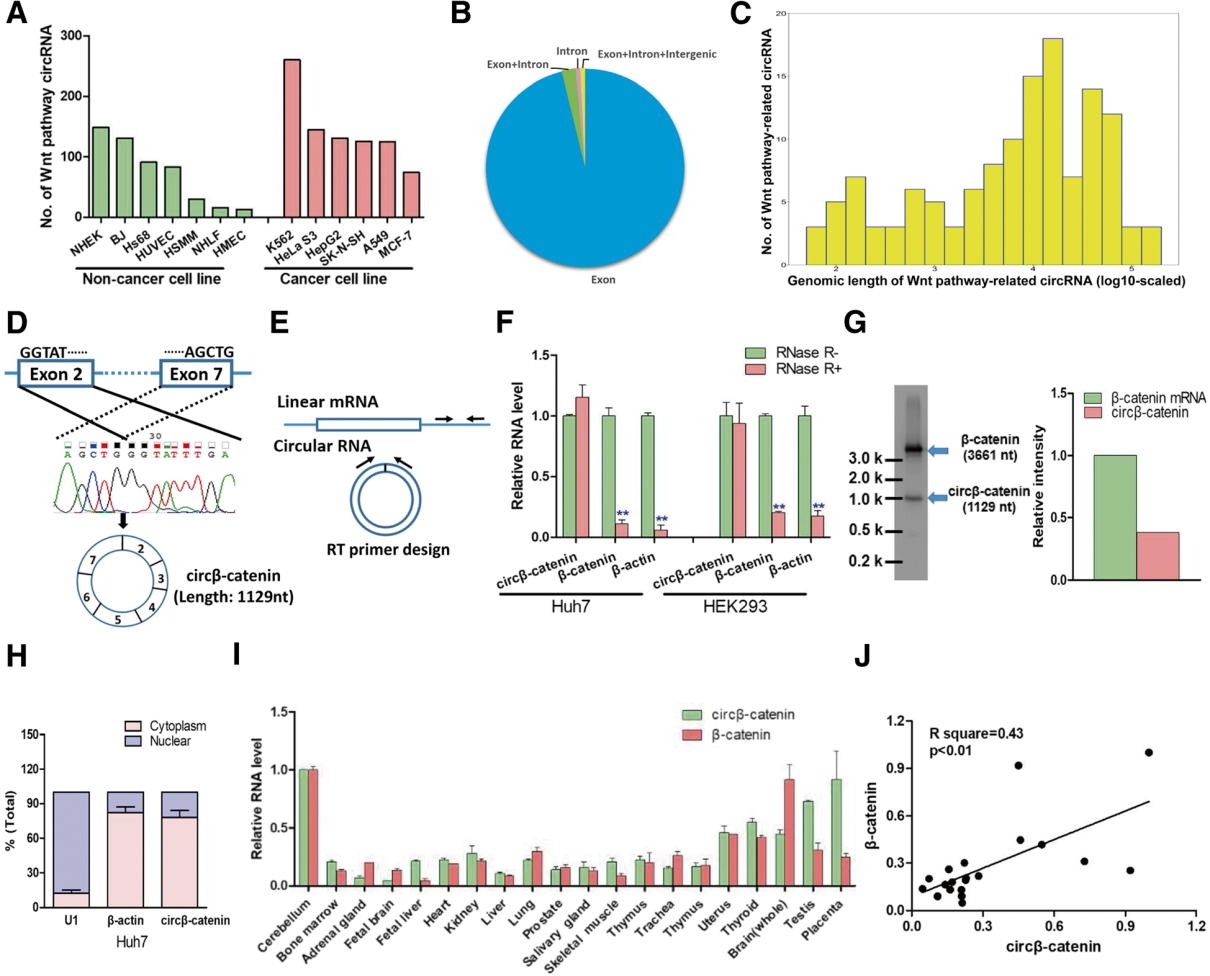
Characterization of circβ-catenin in human cell lines and tissues. **a** The number of Wnt pathway-related circRNAs in human cell lines. **b** Genomic origin of Wnt pathway-related circRNAs in HepG2 cells. **c** The length distribution of Wnt pathway-related circRNAs in HepG2 cells. **d** Schematic illustration of the structure of circular RNA circβ-catenin. Sanger sequencing was conducted to confirm head-to-tail splicing. **e** Convergent and divergent primers were designed to detect linear and circular β-catenin RNAs, respectively. **f** Circβ-catenin was resistant to RNase-R treatment (*n* = 4). **g** Northern blotting was conducted to evaluate the RNA level of circular RNA and linear mRNA for β-catenin in PLC/PRF/5 cells. **h** The expression of circβ-catenin was assessed by qRT-PCR in the nuclear and cytoplasm fractions (*n* = 4). **i** The RNA levels of circβ-catenin and β-catenin mRNA were examined across 20 human tissues using qRT-PCR. **j** The correlation of circβ-catenin and β-catenin mRNA in 20 human tissues (**P < 0.01)

In terms of the pathological importance of Wnt/β-catenin pathway in hepatocarcinogenesis, we selected 143 Wnt pathway-related genes and examined how many circular RNAs were derived from these genes by analyzing the circular RNA sequencing data from online database circBase (Additional file 1: Table S1). We calculated the number of Wnt pathway-related circRNAs from seven non-cancer cell lines and six cancer cell lines and found that most of them can generate hundreds of Wnt pathway-related circRNAs (Fig. 1a). We next focused on the HepG2 cell line in terms of its hepatic origin. We found that 48 Wnt pathway-related genes produced 131 circRNAs in HepG2 cells (Additional file 1: Table S1). Consistent with previous reports [13, 15], the majority of circRNAs comes from exon regions (Fig. 1b) and the genomic length of most exonic circRNAs are more than 1000 nucleotides (Fig. 1c).

### Characterization of circβ-catenin in human cells and tissues

Among the candidate Wnt pathway-related circRNAs, circ-0004194 originated from β-catenin gene locus was chosen for further investigation because (1) β-catenin is a master regulator of Wnt pathway in liver cancer, (2) this circRNA was the only circular RNA which was widely detected in three independent sequencing datasets (Additional file 2: Figure S1), and (3) previous studies showed that the copy number of β-catenin gene locus was frequently amplified in liver cancer tissues.

Subsequent sequence analysis displayed that circ-0004194 (termed “circβ-catenin” in this study) was 1129 nucleotides and contained six exons. By using a divergent primer in cDNA samples and Sanger sequencing, we confirmed that the head-to-tail splicing (back-splicing) occurred in the exons from β-catenin (Fig. 1d). We then designed the convergent and divergent primers to detect the linear mRNA and circular RNA, respectively (Fig. 1e). After treatment with RNase-R, linear β-catenin mRNA displayed a significant reduction whereas circβ-catenin was resistant to RNase-R digestion (Fig. 1f), suggesting that circβ-catenin was more stable than its linear cognate mRNA. Northern blotting was also performed to monitor the RNA levels of circβ-catenin and linear β-catenin mRNA (Fig. 1g). Subsequent qRT-PCR analysis of cell fractions showed that circβ-catenin predominantly localized in the cytoplasm rather than nuclear (Fig. 1h). We also examined the expression profiles of circβ-catenin and β-catenin mRNA across various human tissues (Fig. 1i). We found that the expression of circβ-catenin was positively correlated with linear β-catenin mRNA in human tissues (Fig. 1j).

### Knockdown of circβ-catenin repressed liver cancer cell growth and migration

**Fig. 2:**
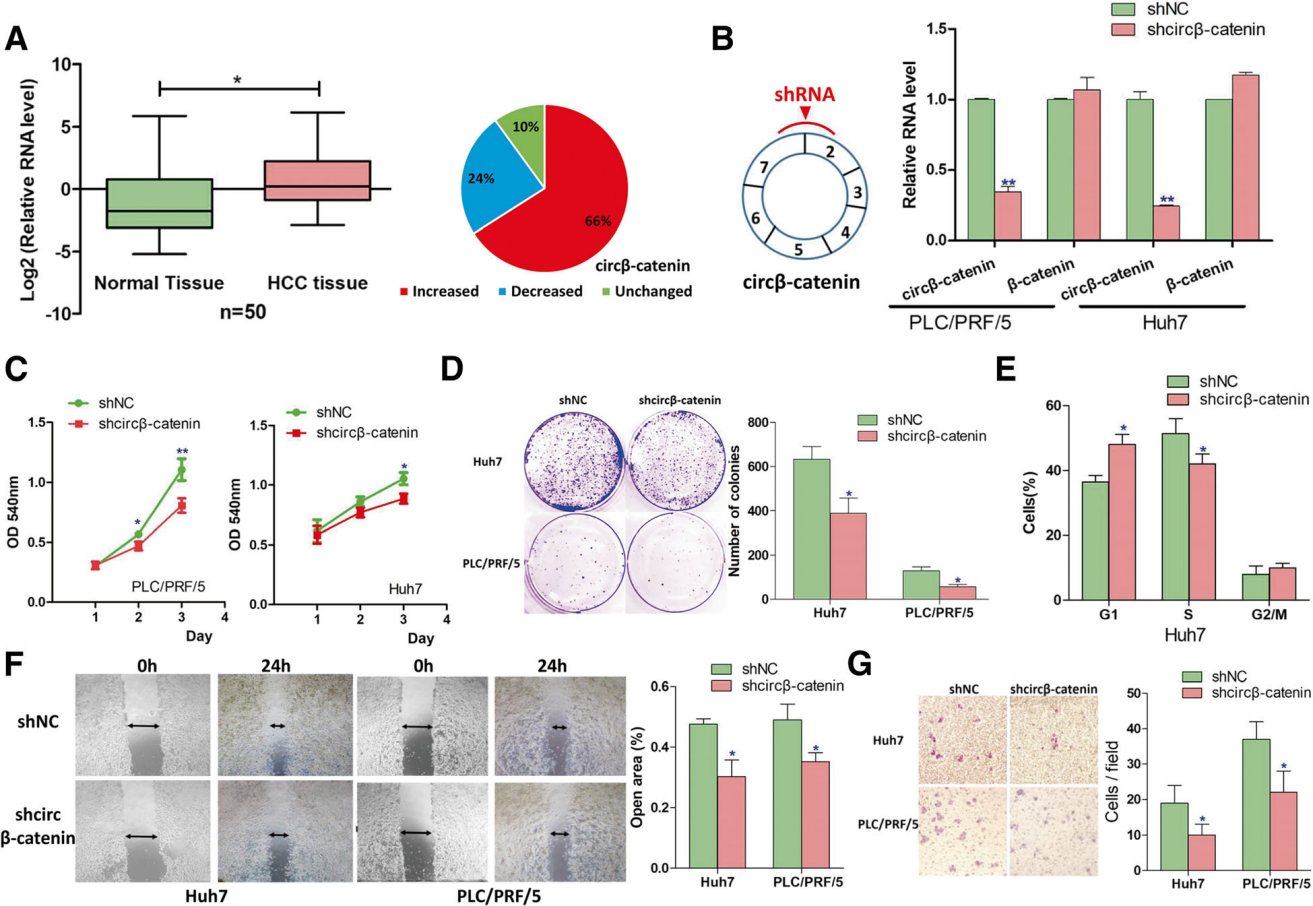
Knockdown of circβ-catenin suppressed in vitro liver cancer cell proliferation and migration. **a** The RNA levels of circβ-catenin were examined in HCC patients (*n* = 50). **b** After stable knockdown of circβ-catenin, the RNA levels of circβ-catenin and β-catenin mRNA were examined in two liver cancer cell lines. **c** MTT assay was performed to monitor the effect of circβ-catenin in cell proliferation (*n* = 6). **d** After stable knockdown of circβ-catenin, colony formation assay was conducted (*n* = 5). **e** The effect of circβ-catenin in modulating cell cycle progression was evaluated by flow cytometry assay (*n* = 5). **f** The effect of circβ-catenin on cell migration was examined by wound healing assay (*n* = 5). **g** The effect of circβ-catenin on cell invasion was examined by Boyden chamber assay (*n* = 4) (*P < 0.05, **P < 0.01)

In order to evaluate the biological importance of circβ-catenin, we then analyzed the expression of circβ-catenin in liver cancer tissues and found that circβ-catenin was significantly increased in liver cancer tissues (Fig. 2a). Furthermore, circβ-catenin was also highly expressed in most of the liver cancer cell lines when compared to the normal liver cell line LO2 (Additional file 2: Figure S2). In order to further characterize the pathological function of circβ-catenin in liver cancer, we generated shRNA viral vector specifically targeted circβ-catenin and found that the shRNA vector stably silenced the RNA levels of circβ-catenin in two liver cancer cell lines (Fig. 2b). However, knockdown of circβ-catenin did not affect the mRNA level of β-catenin (Fig. 2b). We then monitored the effect of circβ-catenin in modulating cancer cell growth. The MTT assay (Fig. 2c), colony formation assay (Fig. 2d), and flow cytometry assay (Fig. 2e) demonstrated that knockdown of circβ-catenin significantly suppressed liver cancer cell growth as well as cell cycle progression. The wound healing assay showed that silencing of circβ-catenin inhibited cancer cell migration (Fig. 2f). Furthermore, the invasion assay showed that knockdown of circβ-catenin also impaired cell invasion capacity (Fig. 2g).

We next conducted rescue experiments to examine whether the phenotypes induced by knockdown of circβ-catenin can be rescued. Based on the results from MTT assay, colony formation assay, and wound healing assay, we found that shRNA-mediated phenotypes can be rescued by ectopic expression of circβ-catenin (Additional file 2: Figure S3). In addition, it is possible that these cellular phenotypes might result from the non-coding function of circβ-catenin. To further clarify this issue, we thus tried to rescue the phenotypes using circβ-catenin expression vectors with a mutated start codon. However, the mutated expression vector fails to rescue the shRNA-mediated phenotypes (Additional file 2: Figure S4). These data might indicate that the cellular phenotypes largely reply on its protein-coding capacity rather than its non-coding function.

### Knockdown of circβ-catenin attenuated in vivo tumorigenesis and metastasis

**Fig. 3:**
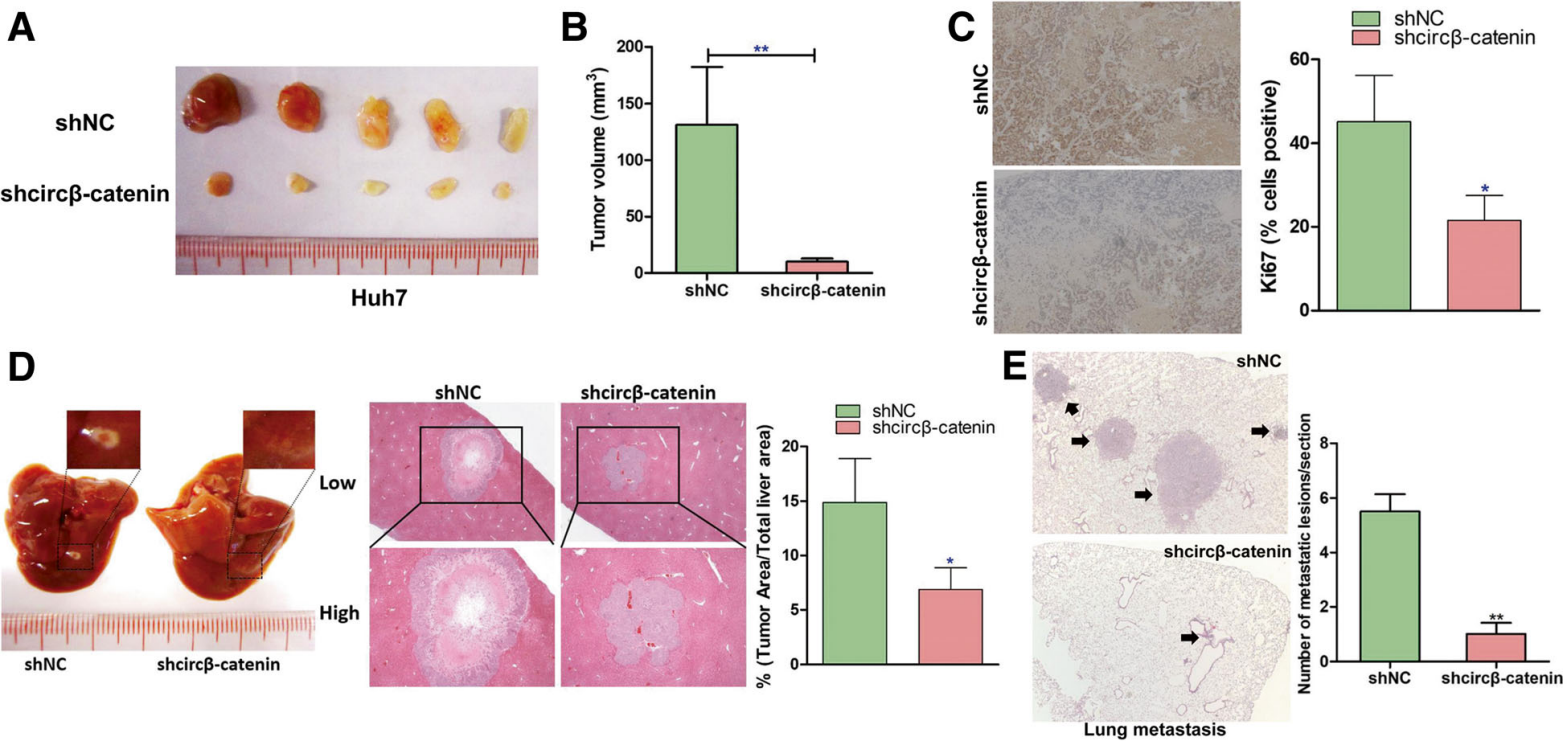
Knockdown of circβ-catenin attenuated in vivo liver cancer cell growth and metastasis. **a** The liver cancer cells with stable knockdown of circβ-catenin were subcutaneously injected into nude mice, and the tumor tissues were collected at indicated time points (*n* = 5). **b** Tumor volume was measured and calculated (*n* = 5). **c** Tumor tissues were stained with Ki67 antibody, and the percentage of Ki67-positive cell was quantified by ImageJ. **d** Huh7 cells with stable knockdown of circβ-catenin were transplanted into the liver and the tumor size was observed after H&E staining (*n* = 5). The percentage of tumor area was quantified by ImageJ. **e** By using Huh7 cell line, lung metastasis model was established to examine the effect of circβ-catenin in modulating cancer metastasis (*n* = 5) (*P < 0.05, **P < 0.01)

To further confirm the in vitro findings, we then verified the biological role of circβ-catenin in mediating in vivo tumorigenicity. The Huh7 cancer cells with stable knockdown of circβ-catenin were subcutaneously implanted into nude mice. Consistent with previous findings, the silence of circβ-catenin dramatically attenuated tumor growth (Fig. 3a, b and Additional file 2: Figure S5). We then monitored cancer cell proliferation by using Ki67 staining, a marker of proliferating cells. Weaker Ki67 staining was found in the tumor tissues with stable knockdown of circβ-catenin when compared to control group (Fig. 3c). Moreover, in terms of that the subcutaneous model does not faithfully recapitulate the microenvironment of liver cancer, we applied liver orthotopic models and found that orthotopic transplantation of Huh7 cells with stable knockdown of circβ-catenin displayed smaller tumor size when compared to control group (Fig. 3d). Furthermore, to evaluate the effect of circβ-catenin on cancer metastasis, a metastasis animal model was generated by injecting liver cancer cells through the tail vein. Less metastatic lesions were found in the lungs of nude mice which were injected with Huh7 cells with stable knockdown of circβ-catenin (Fig. 3e).

### Silence of circβ-catenin inhibited Wnt/β-catenin pathway

**Fig. 4:**
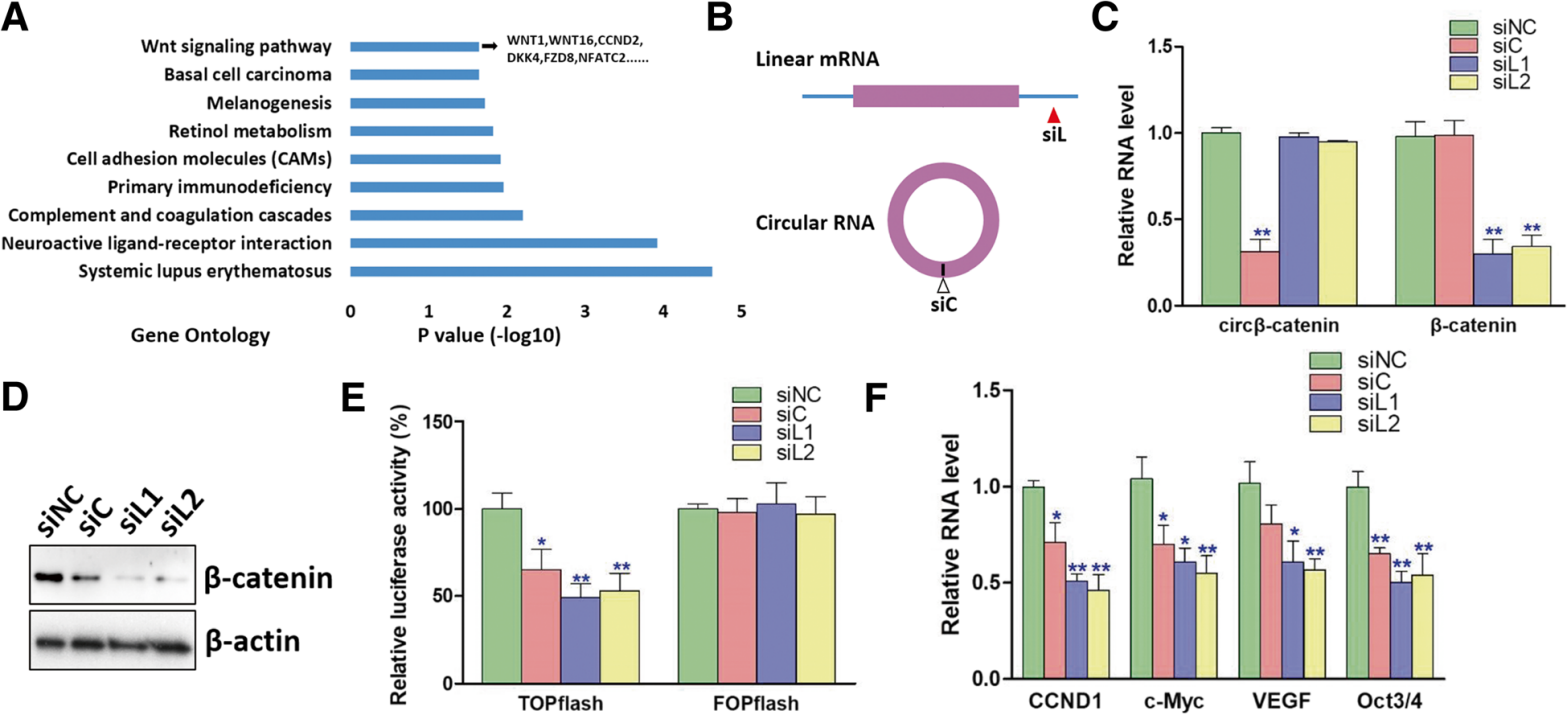
Knockdown of circβ-catenin inhibited Wnt/β-catenin pathway. **a** After stable knockdown of circβ-catenin in PLC/PRF/5 cell line, gene ontology was conducted to analyze the pathways modulated by circβ-catenin. **b** Schematic illustration of the siRNAs targeting circβ-catenin or linear β-catenin mRNA. **c** The RNA levels of circβ-catenin and β-catenin mRNA were analyzed after knockdown of circβ-catenin or β-catenin mRNA in PLC/PRF/5 cells (*n* = 4). **d** The protein levels of β-catenin were analyzed after knockdown of circβ-catenin or β-catenin mRNA in PLC/PRF/5 cells. **e** The luciferase activities of TOPflash and FOPflash were measured after knockdown of circβ-catenin or β-catenin mRNA in PLC/PRF/5 cells (*n* = 4). **f** The expression of the β-catenin target gene was monitored by qRT-PCR in PLC/PRF/5 cells (*n* = 4) (*P < 0.05, **P < 0.01)

As β-catenin is an oncogenic transcription factor in liver cancer, we suspected that circβ-catenin might also affect global RNA transcription directly or indirectly. To shed light on the underlying molecular mechanisms of circβ-catenin in liver cancer, we analyzed the global RNA expression profiles after knockdown of circβ-catenin. Interestingly, bioinformatics analysis of the RNA-seq data indicated that the genes affected by circβ-catenin were significantly enriched in the Wnt/β-catenin pathway (Fig. 4a and Additional file 3: Table S2). The sequencing data was also confirmed by RT-PCR examination (Additional file 2: Figure S6). We then synthesized different siRNAs targeting circβ-catenin or β-catenin mRNA (Fig. 4b). Although siRNA against circβ-catenin did not affect the mRNA level of β-catenin (Fig. 4c), it significantly reduced the protein level of β-catenin (Fig. 4d). A subsequent study showed that siRNAs targeting circβ-catenin significantly reduced the luciferase activity of TOPflash vector harboring β-catenin binding sites while they did not alter the luciferase activity of FOPflash vector with mutated β-catenin binding sites (Fig. 4e). In addition, the siRNAs targeting circβ-catenin or β-catenin also suppressed the RNA expression of several β-catenin target genes (Fig. 4f).

### Circβ-catenin encoded a novel β-catenin isoform

**Fig. 5:**
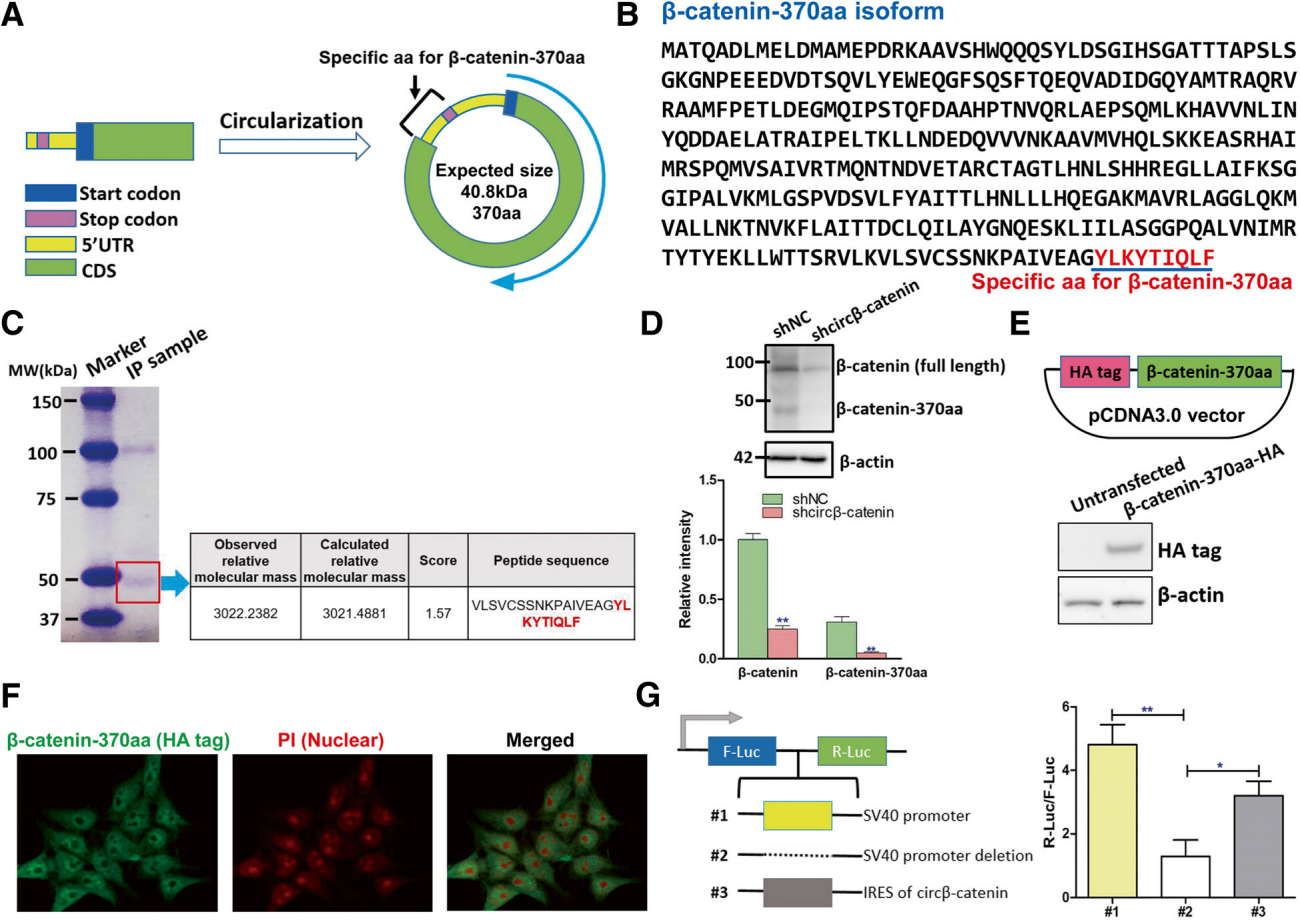
Circβ-catenin has the protein-coding capacity. **a** Schematic illustration of the circularization of circβ-catenin. **b** The predicted sequence of β-catenin-370aa. **c** After pulled down by using a β-catenin antibody, the protein samples from PLC/PRF/5 cells were subject to mass spectrometry analysis. And the specific peptides from β-catenin-370aa were identified. **d** The expression levels of full-length β-catenin and β-catenin-370aa were examined by western blotting after stable knockdown of circβ-catenin in PLC/PRF/5 cells. **e** Validation of β-catenin-370aa with HA tag by western blotting. **f** Immunostaining was conducted to examine the cellular location of β-catenin-370aa in HEK293 cells. **g** The putative IRES activity in circβ-catenin was tested by Dual-Luciferase Reporter Assay (*P < 0.05, **P < 0.01)

Emerging evidence indicated some circular RNAs have the protein-coding capacity, we next analyzed the putative open reading frame in circβ-catenin. The bioinformatics analysis showed that circβ-catenin had putative internal ribosome entry site (IRES) sequence and it might encode a novel β-catenin isoform with 370 amino acids (Fig. 5a and Additional file 2: Figure S7), which was termed “β-catenin-370aa” in this study. The expected size of this isoform was 40.8 kDa. This novel isoform shared homologous N-terminus sequence with wild type β-catenin, but it contained a new C-terminus with 9 specific amino acids (Fig. 5b). To confirm the existence of this novel isoform, we utilized the mass spectrometer. We first used an antibody which specifically recognized the N-terminus of β-catenin to conduct co-immunoprecipitation, which can enrich the β-catenin protein isoforms with complete N-terminus sequence. The mass spectrometry analysis successfully identified the specific peptide fragments from β-catenin-370aa (Fig. 5c and Additional file 4: Table S3). We then performed the western blotting and found that this antibody can recognize several β-catenin isoforms and knockdown of circβ-catenin reduced the protein levels of full-length β-catenin and β-catenin-370aa (Fig. 5d).

Next, we inserted the full-length sequence of β-catenin-370aa into a plasmid with HA tag (Fig. 5e). The immunostaining results displayed that this protein was mainly located in the cytoplasm (Fig. 5f). Moreover, to examine the putative IRES activity in circβ-catenin, we used a Dual-Luciferase Reporter System. This system has Renilla luciferase (R-Luc) and Firefly luciferase (F-Luc) with independent promoters. We first deleted the promoter of R-Luc and then inserted the 107 bp putative IRES sequence into the region in front of R-Luc gene (Fig. 5g). We found that the deletion of the promoter from R-Luc significantly reduced the luciferase activity of R-Luc while insertion of IRES sequence of β-catenin partially restored the luciferase activity (Fig. 5g), suggesting that IRES of circβ-catenin might be useful in initiating gene translation.

### β-Catenin-370aa protected β-catenin from GSK3β-mediated degradation

**Fig. 6:**
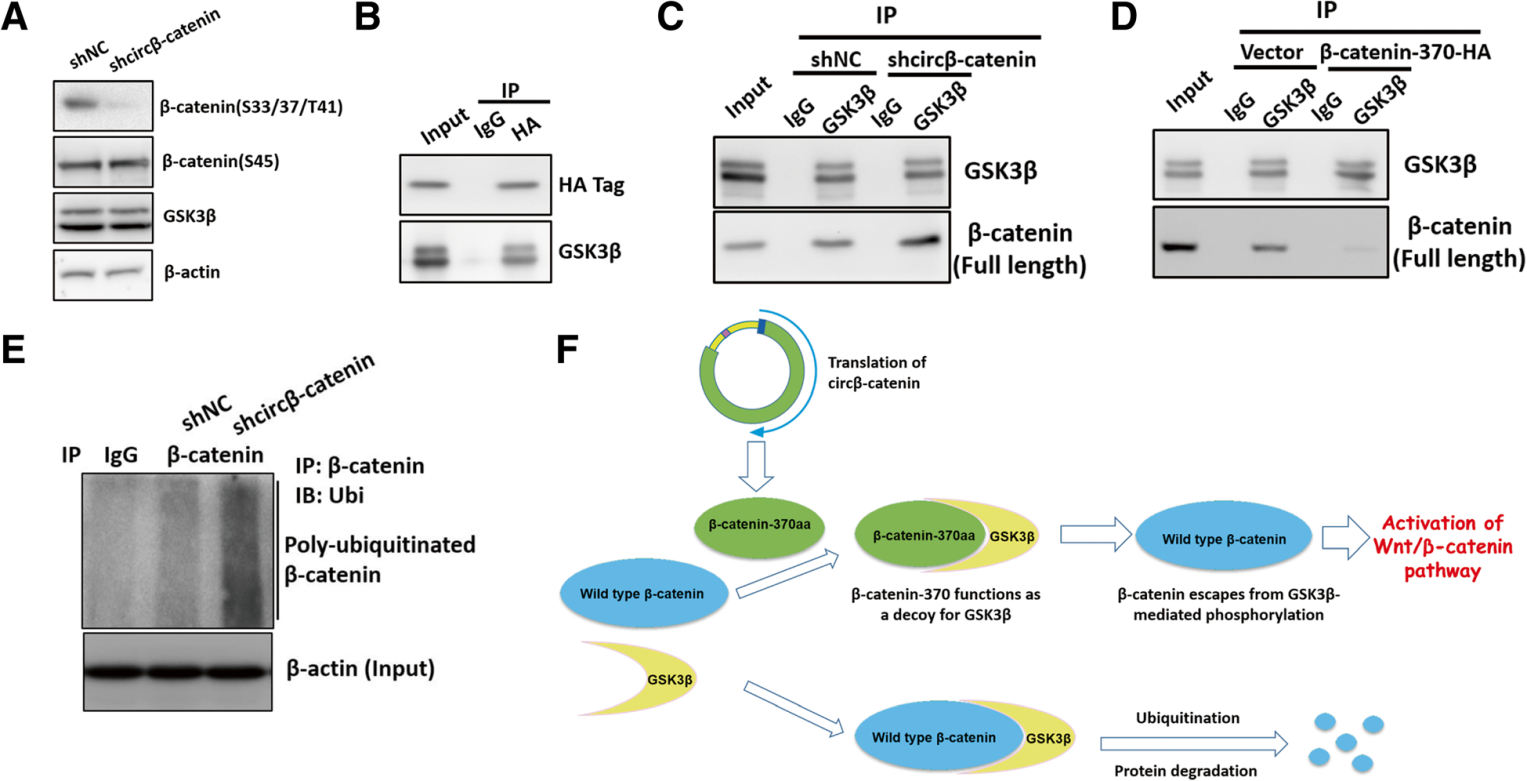
β-catenin-370aa functioned as a decoy for GSK3β and antagonized GSK3β-mediated β-catenin degradation. **a** The phosphorylation status of β-catenin was examined by western blot in Huh7 cells. **b** IP experiment was performed to evaluate the interaction between β-catenin-370aa and GSK3β in Huh7 cells. **c** The binding capacity between β-catenin and GSK3β was monitored by co-IP in circβ-catenin knockdown Huh7 cells and corresponding control cells. **d** After transient overexpression of β-catenin-370aa, the binding capacity between β-catenin and GSK3β was monitored by co-IP. **e** In liver cancer cells with stable knockdown of circβ-catenin, western blot was conducted to examine the endogenous β-catenin-associated ubiquitination after immunoprecipitation with anti-β-catenin antibody in Huh7 cells. **f** Schematic diagram of a hypothetical model

Because silence of circβ-catenin suppressed the protein level of β-catenin without affecting the RNA level of β-catenin, we thus speculated that circβ-catenin might modulate the expression of β-catenin in protein level rather than the transcription level. Previous studies demonstrate that the stability of β-catenin is tightly linked with its phosphorylation status. Upon phosphorylation by GSK3β at three residues (Ser33/Ser37/Thr41), β-catenin is ubiquitinated by ubiquitin ligase β-TrCP and subsequently degraded by the proteasome. Therefore, we examined the phosphorylation status of β-catenin. We found that silencing of circβ-catenin decreased the phosphorylation at Ser33/Ser37/Thr41 sites (Fig. 6a). In terms of that GSK3β could interact with the N-terminus of β-catenin, we suspected that GSK3β might physically interact with β-catenin-370aa, which also harbors part of the N-terminal sequence of β-catenin. According to the co-IP results, as expected, we showed that GSK3β physically interacted with β-catenin-370aa (Fig. 6b).

We next evaluated the effect of circβ-catenin in modulating the mutual interaction between GSK3β and full-length β-catenin. Based on the co-IP results, we found that silencing of circβ-catenin enhanced the interaction between GSK3β and full-length β-catenin (Fig. 6c) whereas ectopic expression of β-catenin-370aa impaired the interaction between GSK3β and full-length β-catenin (Fig. 6d). Furthermore, we also found that ectopic expression of β-catenin-370aa can potentiate Wnt/β-catenin pathway (Additional file 2: Figure S8). Given that GSK3β-mediated phosphorylation of β-catenin might trigger β-catenin ubiquitination, we utilized β-catenin antibody to pull down endogenous β-catenin proteins after knockdown of circβ-catenin and subsequently examined their modification by ubiquitin antibody. According to the co-IP results, we detected an increased β-catenin ubiquitination after knockdown of circβ-catenin (Fig. 6e), suggesting that circβ-catenin might strengthen β-catenin stability by reducing its ubiquitination. Taken together, these findings indicated that β-catenin-370aa encoded by circβ-catenin might function as a decoy for GSK3β (Fig. 6f), leading to escape from GSK3β-induced β-catenin degradation.

## Discussion

In the past decades, numerous investigations have displayed abnormal expression profiles of non-coding RNAs in a wide spectrum of cancer types. Recent studies have unraveled that many miRNAs and lncRNAs played vital roles in modulating tumor growth and cancer metastasis [4, 5]. Nonetheless, how circRNAs participate in cancer progression remains elusive [26, 27]. Nowadays, only a few circRNAs have been identified to play a role in liver cancer [28]. In the current study, we characterized a previously unknown circular RNA circβ-catenin and evaluated its functional role in modulating liver cancer growth. We found that circβ-catenin was highly expressed in liver cancer tissues and stable knockdown of circβ-catenin significantly suppressed malignant phenotypes of liver cancer cells. Subsequent studies demonstrated that circβ-catenin was translatable and it generated a novel isoform named β-catenin-370aa. We found that β-catenin-370aa physically interacted with GSK3β, which can phosphorylate β-catenin and thus trigger the degradation of β-catenin. β-catenin-370aa competitively interacted with GSK3β and served as a decoy that prevented GSK3β from binding to full-length β-catenin, leading to antagonization of GSK3β-induced β-catenin degradation, which might shed light on the protein-coding potential of circRNAs during tumor progression.

In the current study, we find that β-catenin-370aa serves as a decoy and competitively prevents binding of GSK3β to full-length β-catenin protein. According to the results from western blot, we find that β-catenin-370aa is around 3-fold less abundant than the full-length β-catenin protein (Fig. 5d). We also find that silence of the circβ-catenin significantly reduces the full-length β-catenin protein (Fig. 5d). These results indicate that β-catenin-370aa is an effective competitor. This effect might be related to its cellular localization. It is well characterized that active full-length β-catenin is mainly localized in the nucleus, and only a small portion of them stays in the cytoplasm. In our study, we find that β-catenin-370aa is predominantly localized in the cytoplasm (Fig. 5f). Due to cellular co-localization with GSK3β in the cytoplasm, more β-catenin-370aa protein may interact with GSK3β when compared to full-length β-catenin protein. Therefore, β-catenin-370aa is an effective competitor, and perhaps, this effect is related to its cellular localization.

Alternative splicing of linear mRNA provides the potential to generate a large number of diverse protein isoforms from limited protein-coding genes within the human genome [29]. Intriguingly, emerging evidence indicates that a few circular RNAs can produce novel protein isoforms through back-splicing and circularization of selected exons from their linear mRNA transcripts. After circularization, novel stop codons are generated and located at the upstream of the start codon AUG in linear mRNAs. Novel protein isoforms with additional amino acids would be produced after translation of circRNAs. Previous investigation showed that 735 circRNAs harbored canonical start codon as their linear counterparts [25]. However, it remains unknown whether they also have stop codon. Therefore, it is particularly interesting to investigate whether these circRNAs have the protein-coding capacity. Furthermore, these intriguing mechanisms provide novel insights into how novel protein isoforms are generated through a pathway independent of conventional alternative splicing, suggesting that the number of protein isoforms from our human genome might be much more than we have expected.

It was recently reported that artificial circRNA can be translated independently of the IRES sequence [21, 30]. Emerging evidence shows that the so-called “non-coding RNA” actually has the protein-coding capacity and they are translatable. For instance, a muscle-specific lncRNA Dworf can encode an endogenous peptide with 34 amino acids, and this peptide can enhance calcium uptake as well as myocyte contractility [31]. In addition, the plant primary miR171b and miR165a transcripts cannot only generate mature miRNAs but also encode regulatory peptides, resulting in delayed root development [32]. Besides lncRNA and miRNA transcripts, a few circRNAs were reported to have a protein-coding function. A muscle-enriched circular RNA circ-ZNF609 was directly associated with polysomes and then translated into a novel ZNF609 protein isoform. The subsequent functional study showed that circ-ZNF609 can control myoblast proliferation [25]. In glioblastoma, a circRNA termed circ-FBXW7, which contains an open reading frame, was frequently downregulated in glioblastoma sample. Circ-FBXW7 could encode a 21-kDa protein which inhibited in vitro and in vivo glioma cell growth [23]. These circRNAs provide a paradigm of a protein-coding circRNA in mammals.

## Conclusion

In conclusion, herein, we identify a novel circRNA derived from β-catenin, a well-characterized oncogene in liver cancer. This is the first report that circRNA can encode a novel protein isoform, which subsequently promotes tumor growth through activation of the Wnt/β-catenin pathway in liver cancer. These findings expand our current knowledge of circRNAs and indicate that the protein-coding capacity of non-coding RNAs especially circRNAs is largely underestimated.

## Methods

### RNA extraction, RNase-R treatment, and real-time PCR

The total RNA was isolated using the FavorPrep™ Tissue Total RNA Mini Kit (Favorgen, Hong Kong). RNase-R treatment was conducted by adding 2U RNase-R (Epicenter, USA) per microgram of RNA, followed by a 1-h incubation at 37 °C. The RNA samples were reversely transcribed into cDNA by High-Capacity cDNA Reverse Transcription Kit (Applied Biosystems, USA). RPLPO gene was used as a reference gene, and the relative fold changes of indicated genes were calculated by 2^−ΔΔCT^ method. The primer sequences were provided in Additional file 5: Table S4.

### Western blot

The indicated cells were lysed by RIPA buffer and subsequently resolved on 10% SDS-PAGE. The membranes were probed by the following antibodies against β-catenin (#9581, Cell Signaling Technology), Phospho-β-Catenin-Ser45 (#9564, Cell Signaling Technology), Phospho-β-Catenin-Ser33/37/Thr41 (#9561, Cell Signaling Technology), GSK3β (MA3-038, ThermoFisher), HA tag (sc-57592, Santa Cruz Biotechnology), and β-actin (sc-47778, Santa Cruz Biotechnology). The expression levels were visualized under enhanced chemiluminescence.

### Sanger sequencing

The cDNA from Huh7 cells were amplified by PCR using a primer specifically targeting circβ-catenin. To confirm the head-to-tail splicing, Sanger sequencing was conducted by using sequencing primer 5′-GAAAAGCGGCTGTTAGTCA-3′.

### Cell culture and lentivirus transduction

The SW620, Huh7, HepG2, and PLC/PRF/5 cell lines were maintained in Dulbecco’s modified Eagle medium (DMEM) supplemented with 10% FBS. The parallel DNA sequences for shRNA were synthesized and cloned into pGLV31H1-GFP vector. The cancer cells (PLC/PRF/5, Huh7, and SW620) with stable knockdown of circβ-catenin were generated by using lentivirus vector pGLV31H1-GFP.

### Plasmid construction

The short hairpin RNA (shRNA) targeting circβ-catenin was designed by online software (http://sirna.wi.mit.edu/). The parallel DNA sequences were synthesized and cloned into pGLV31H1-GFP vector. The human circβ-catenin sequence was copied from human pcDNA3-β-catenin (Addgene #16828) and then inserted into pcDNA3.1 (+) circRNA mini vector (Addgene #60648) using the EcoRV and SacII sites. The sequence of the back-splicing junction has been modified when designing the primers, and thus, the back-splicing junction is not targeted by the shRNA. The overexpression effect was monitored by RT-PCR. The circβ-catenin expression vector with mutated ATG was generated by the Quick Change site-directed mutagenesis kit (Agilent Technologies). The primer sequences for plasmid construction were provided in the Additional file 5: Table S4.

### Colony formation assay

The cancer cells with stable knockdown of circβ-catenin and parallel control cells were plated into 6- or 12-well plates. The cells were maintained in the incubator for 10 days and then visualized by crystal violet staining. The number of colonies was counted by ImageJ software (National Institutes of Health, USA).

### Flow cytometry analysis

The cancer cells with stable knockdown of circβ-catenin and corresponding control cells were fixed with 70% ethanol at 4 °C overnight and then stained with staining buffer (50 μg PI/mL, 50 μg RNase/mL, 0.37 mg/ml EDTA, 1% Triton X-100) at 4 °C for half hour. After PI staining, the cells were subject to flow cytometry analysis using LSR Fortessa and FACSDiva software (BD Biosciences, USA).

### Wound healing assay

The liver cancer cells with stable knockdown of circβ-catenin and their corresponding control cells were grown to 100% confluence. Then, the pipette tips were utilized to scratch the monolayer cells. Twenty-four hours later, the open area was captured and calculated using ImageJ software (National Institutes of Health, USA).

### Cell invasion assay

Transwell assay was conducted in a Boyden chamber with an 8.0-μm pore (Corning, USA), and the upper surface of the chamber was coated with Matrigel. 1 × 10^5^ Huh7 and PLC/PRF/5 cells with stable knockdown of circβ-catenin, and their corresponding control cells were seeded into the upper chamber. Twenty-four hours later, the cancer cells on the upper compartment were removed. Then, the cells were stained with 0.2% crystal violet solution (Sigma-Aldrich, USA) for 15 min. Images were captured, and the cell number was counted by ImageJ (National Institutes of Health, USA).

### Co-immunoprecipitation

The cells were lysed by cold lysis buffer (1% TritonX-100, 50 mM Tris-7.5, 1 mM EDTA, 150 mM NaCl, and protease inhibitors). The supernatant from cell lysates was incubated with indicated antibodies. Then, the protein G beads were incubated with the lysates. The beads were washed by cold lysis buffer, and the protein loading buffer was added to the beads, followed by western blotting analysis.

### RNA sequencing

The total RNA from PLC/PRF/5 cells with stable knockdown of circβ-catenin and control cells were isolated by Trizol (Invitrogen, Hong Kong) following the manufacturer’s protocol. The transcripts were sequenced using the BGISEQ-500 sequencing platform (BGI Tech Company, China). After rRNA depletion, the RNA fragments were reversely transcribed into cDNA by using N6 random primer. The 5′ end of cDNA was phosphorylated, and 3′ end of cDNA was modified with stickiness “A.” Then, the cDNA was ligated with the adapter with sticky “T” at the 3′ end. Two specific primers are utilized to amplify the ligation DNA products. The DNA products were denatured by heat, and the single-stranded DNA was circularized by DNA ligase. Finally, the prepared DNA library was sequenced. Bowtie2 software was used to map clean reads to the reference gene, and HISAT software was utilized to map to the reference genome. Genes with a log_2_ fold change ≥ 1 were considered differentially expressed. The gene ontology analysis was carried out by online program DAVID (https://david.ncifcrf.gov/).

### Mass spectrometry analysis

Mass spectrometry analysis was conducted by LTQ Velos Dual-Pressure Ion Trap Mass Spectrometer (Thermo Scientific, USA). An antibody which specifically recognized the N-terminus of β-catenin (#9581, Cell Signaling Technology) was used to conduct a co-IP experiment to enrich the target proteins. After SDS-PAGE, the whole proteins were digested by using trypsin. Tandem mass spectrometry was used for protein sequencing following the manufacturer’s protocol. The identification of protein fragment was analyzed by the MASCOT database.

### Northern blotting

Digoxin-labeled RNA probe targeting the exon region shared by circβ-catenin and β-catenin was prepared with DIG Northern Starter Kit (Roche, USA). The target sequence of the RNA probe is 5′-GACACGCTATCATGCGTTCTCCTCAGATGGTGTCTGCTATTGTACGTACC-3′. Northern blotting assay was conducted by using Northern Max Kit from Ambion (Life Technologies) by following the manufacturer’s instructions. Electrophoresis of 15 μg total RNA was carried out on a 2% agarose gel and transferred to a Hybond-N^+^ membrane (GE Healthcare). Membranes were subsequently dried and crosslinked by ultraviolet. Pre-hybridization was performed at 62 °C for 2 h, and hybridization was performed at 62 °C for 16 h. The membrane was washed by SSC buffer with 0.1% SDS at 62 °C. After washing, the blots were visualized by the Typhoon scanner (GE Healthcare).

### Bioinformatics analysis

The circular RNA sequence data of Wnt pathway-related genes was obtained from database circBase (http://www.circbase.org/). The protein-coding capacity of circRNA was evaluated by using the online program http://202.195.183.4:8000/circrnadb/circRNADb.php. The predicted protein size was obtained from the website http://www.sciencegateway.org/tools/proteinmw.htm.

### Animal study

For the xenograft tumor model, 2 × 10^7^ Huh7 cells were subcutaneously implanted into five male nude mice. One month later, the nude mice were sacrificed and the tumor tissues were collected. And the tumor volume was calculated via the formula as follows: volume = (width^2^ × length)/2. For the in vivo lung metastasis experiments, 1 × 10^7^ Huh7 cells were introduced into nude BALB/c mice by hydrodynamic tail vein injection. Two months later, the lung tissues were excised and subject to H&E staining to visualize the metastatic lesions within the lung. The lung metastatic lesions were counted under the microscope. For the orthotopic tumor model, the cancer cells were implanted into the liver of male C57BL/6 mice after anesthetization. Forty days later, the liver tissues were collected after the C57BL/6 mice were sacrificed and H&E staining was conducted to visualize the hepatic tumors. The animal handling procedures were approved by the institutional LASEC Guidelines in The Chinese University of Hong Kong. All animals were housed in an enriched environment and can freely access to food and water at 12:12-h light/dark cycle room.

### Luciferase reporter assay

HepG2 cells were transfected with indicated luciferase vectors. After lysed by lysis buffer, the luciferase activity was measured by the Dual-Luciferase® Reporter Assay System (Promega, Hong Kong).

### Clinical tissue sample analysis

Fifty paired HCC tissues and corresponding adjacent normal tissues were obtained from Prince of Wales Hospital (The Chinese University of Hong Kong, Hong Kong) as previously reported [4, 5]. Informed consent was obtained from each patient, and this clinical study was approved by The Joint Chinese University of Hong Kong-New Territories Ease Cluster Clinical Research Ethics Committee.

### Statistics

The data are expressed as mean ± SD. The significance of difference was evaluated using Student’s *t* test. The correlation study was conducted using Pearson’s correlation. When the *P* value is less than 0.05, the results were considered to be statistically significant.

## Additional files

Additional file 1: **Table S1.** A gene list of 143 Wnt pathway-related genes and their corresponding circular RNAs (XLSX 34 kb)

Additional file 2: **Figure S1–S8.** with figure legends (PDF 668 kb)

Additional file 3: **Table S2.** The RNA expression profile for deregulated genes after knockdown of circβ-catenin (XLSX 477 kb)

Additional file 4: **Table S3.** The mass spectrometry analysis data (XLSX 78 kb)

Additional file 5: **Table S4.** The primer and siRNA sequences (PDF 284 kb)
