# Supplementary material for: Translation of the circular RNA circβ-catenin promotes liver cancer cell growth through activation of the Wnt pathway
Source: Genome Biol. 2019 Apr 26;20:84. doi: 10.1186/s13059-019-1685-4 (PMC6486691; doi:10.1186/s13059-019-1685-4)
Supplement: Supplementary file 2 — Figure S1–S8. with figure legends (PDF 668 kb) [file 13059_2019_1685_MOESM2_ESM.pdf]

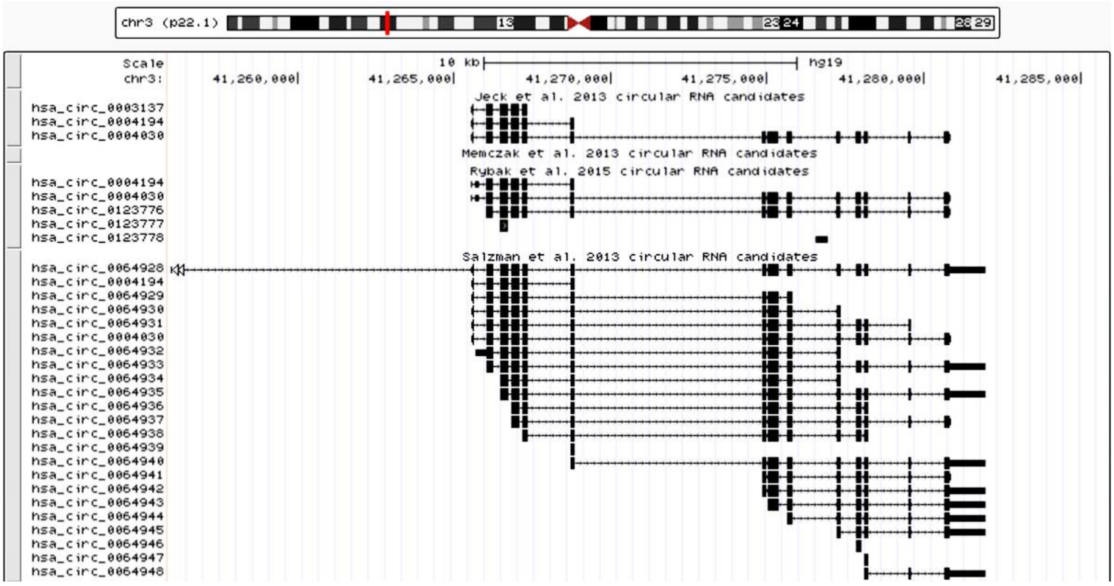

Figure S1 The sequencing data of circβ-catenin from database circBase.

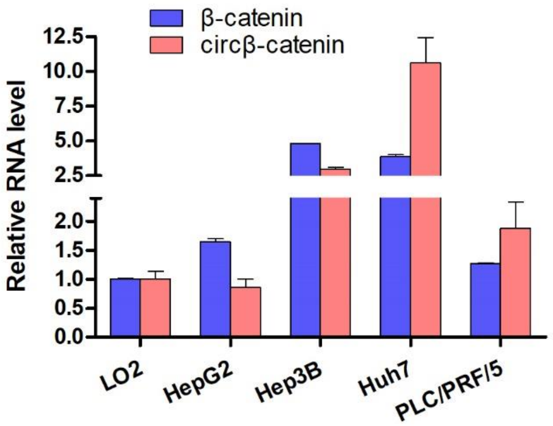

Figure S2 The RNA levels of circβ-catenin were examined in the normal liver cell line LO2 and four liver cancer cell lines. RPLP0 gene was used as reference gene for normalization and its mRNA levels do not change across cell lines.

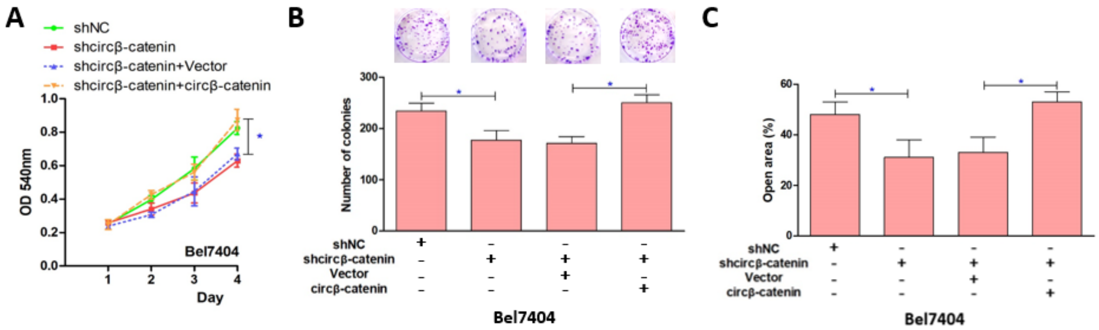

Figure S3 The Bel7404 cells with stable knock down of circβ-catenin were transfected with empty vector or circβ-catenin expression vector. MTT assay (A), colony formation assay (B) and wound healing assay (C) showed that the circβ-catenin overexpression vector could rescue the shRNA-mediated phenotypes. (n=4 ;*, $P < 0.05$)

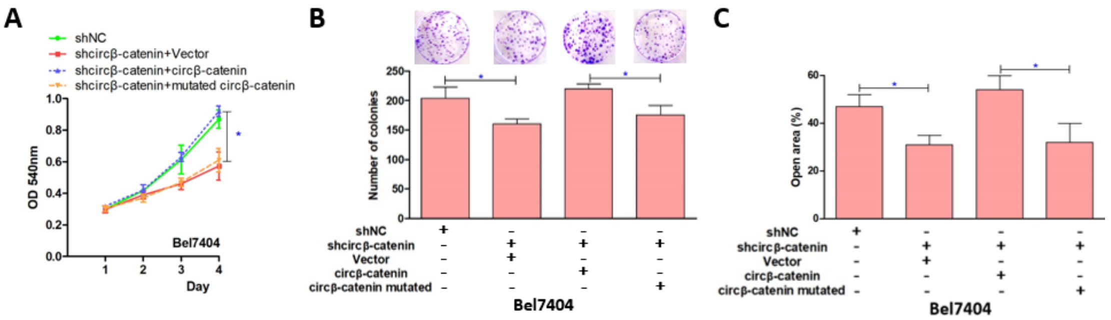

Figure S4 The Bel7404 cells with stable knock down of circβ-catenin were transfected with empty vector, circβ-catenin expression vector or circβ-catenin expression vector with mutated start codon. MTT assay (A), colony formation assay (B) and wound healing assay (C) showed that the circβ-catenin overexpression vector can rescue the shRNA-mediated phenotypes while the mutated expression vector failed to rescue them. (n=4 ;*, $P < 0.05$)

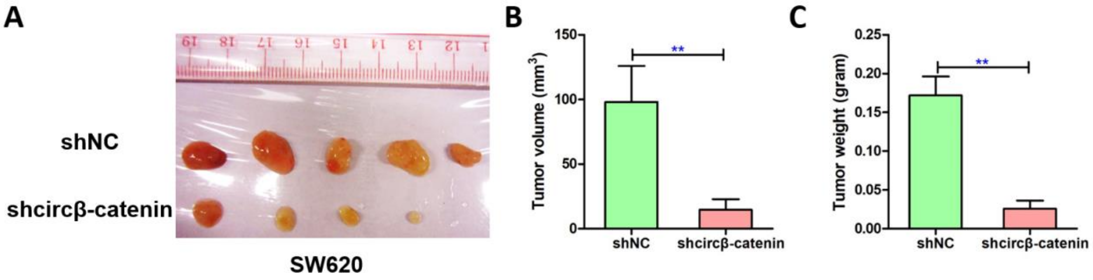

Figure S5 Knockdown of circβ-catenin suppressed *in vivo* tumor growth.

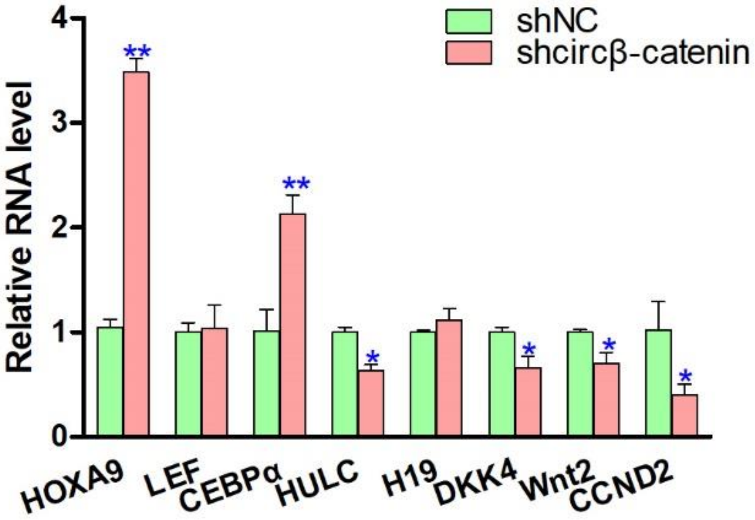

Figure S6 Validation of the sequencing data by using RT-PCR examination.

<table>
<tr><th>Transcript id</th><th colspan="4">Exon information</th></tr>
<tr><td rowspan="2">NM_001904, NM_001098209, NM_001098210</td><td>Spliced_len</td><td>Exon Number</td><td>Exon Sizes</td><td>Exon Offsets</td></tr>
<tr><td>1129</td><td>6</td><td>61, 228, 254, 239, 202, 145</td><td>0, 505, 933, 1313, 1639, 3187</td></tr>
<tr><td colspan="5">Protein coding potential</td></tr>
<tr><td rowspan="4">IRES Elements</td><td colspan="4">Parameter Index</td></tr>
<tr><td colspan="2">Position (start--end)</td><td>R Score</td><td>With Pseudoknot (Y/N)</td></tr>
<tr><td colspan="2">760--866</td><td>1.543406</td><td>Y</td></tr>
<tr><td colspan="2">46--70</td><td>1.366318</td><td>Y</td></tr>
<tr><td rowspan="4">Open Reading Frame (ORF)</td><td colspan="2">Start Position</td><td>End Position</td><td>Protein Length</td></tr>
<tr><td colspan="2">49</td><td>1r+32</td><td>370 aa</td></tr>
<tr><td colspan="4">MATQADLMEL DMAMEPDRKA AVSHWQQQSY LDSGIHSGAT TTAPSLSGKG NPEEEDVDTS QVLYEWEQGF SQSFTQEQVA
DIDGQYAMTR AQRVRAAMFP ETLDEGMQIP STQFDAAHPT NVQRLAEPSQ MLKHAVVNLI NYQDDAELAT RAIPELTKLL
NDEDQVVVNK AAVMVHQLSK KEASRHAIMR SPQMVSAIVR TMQNTNDVET ARCTAGTLHN LSHHREGLLA IFKSGGIPAL
VKMLGSPVDS VLFYAITTLH NLLLHQEGAK MAVRLAGGLQ KMVALLNKTN VKFLAITTDC LQILAYGNQE SKLIILASGG
PQALVNIMRT YTYEKLLWTT SRVLKVLSVC SSNKPAIVEA GYLKYTIQLF *</td></tr>
<tr><td colspan="4">Note:
(1). nr represents n rounds(n<3); (2). * represents a stop codon.</td></tr>
</table>

Figure S7 Evaluation of the coding capacity of circβ-catenin.

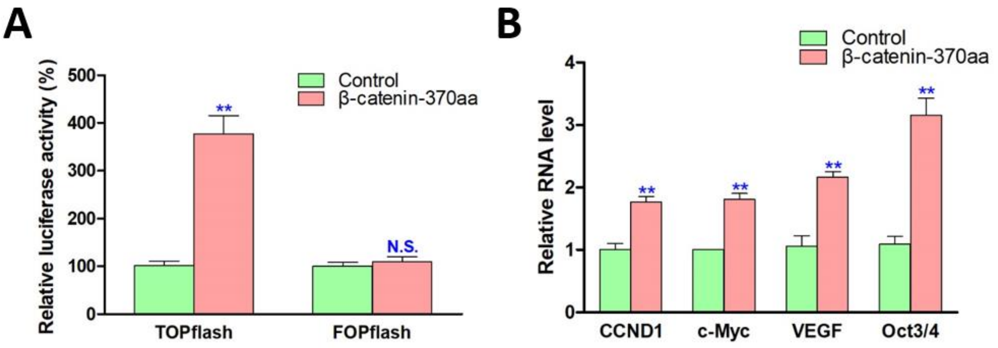

Figure S8 β-catenin-370aa activated Wnt/β-catenin pathway.
