# Supplementary material for: Translation of the circular RNA circβ-catenin promotes liver cancer cell growth through activation of the Wnt pathway
Source: Genome Biol. 2019 Apr 26;20:84. doi: 10.1186/s13059-019-1685-4 (PMC6486691; doi:10.1186/s13059-019-1685-4)
Supplement: Supplementary file 5 — Table S4. The primer and siRNA sequences (PDF 284 kb) [file 13059_2019_1685_MOESM5_ESM.pdf]

**Name**	**Primer sequences used for plasmid construction**
pcDNA3.1-3XHA-β-catenin-370aa-F	CGGAATTCCGCTACTCAAGCTGATTTGAT
pcDNA3.1-3XHA-β-catenin-370aa-R	AAGGTACCTCAAAACAGTTGTATGGTATACTTCAAATACCCAGCTTCTACAATAGCCGG
circβ-catenin-shRNA-F	GATCCGCTGGGTATTTGAAGTATATTCAAGAGATATACTTCAAATACCCAGCTTTTTT
circβ-catenin-shRNA-R	AATTAAAAAAGCTGGGTATTTGAAGTATATCTCTTGAATATACTTCAAATACCCAGCG
circβ-catenin-F	CCGATATCCCGATTTGAAGTATACCATA
circβ-catenin-R	TCCCCGCGGTGACTTCTACAATAGCCGGC
**Name**	**Primer sequences used for RT-PCR**
U1-F	TGATCACGAAGGTGGTTTTCC
U1-R	GCACATCCGGAGTGCAATG
β-actin-F	AAGATGACCCAGATCATGTTTGAG
β-actin-R	GCAGCTCGTAGCTCTTCTCCAG
RPLP0-F	CCGGATATGAGGCAGCAGTT
RPLP0-R	GAAGGCTGTGGTGCTGATGG
β-catenin-F	GGGTCCTCTGTGAACTTGCTC
β-catenin-R	TTCTTGTAATCTTGTGGCTTGTCC
circβ-catenin-F	AGTGCTGAAGGTGCTATCTGT
circβ-catenin-R	AGGTAAGACTGTTGCTGCCA
CCND2-F	ACCTTCCGCAGTGCTCCTA
CCND2-R	CCCAGCCAAGAAACGGTCC
HOXA9-F	TACGTGGACTCGTTCCTGCT
HOXA9-R	CGTCGCCTTGGACTGGAAG
H19-F	TGCTGCACTTTACAACCACTG
H19-R	ATGGTGTCTTTGATGTTGGGC
HULC-F	AACCTCCAGAACTGTGAT
HULC-R	CATAATTCAGGGAGAAAG
Wnt2-F	GATGCGTGCCATTAGCCAG
Wnt2-R	AGATTCCCGACTACTTCGGAG
DKK4-F	ACGGACTGCAATACCAGAAAG
DKK4-R	CGTTCACACAGAGTGTCCCAG
**Name**	**siRNA sequences**
siL1	GCUUGGAAUGAGACUGCUG
siL2	GAAUGCAGUUCGCCUUCAC
siC	GCUGGGUAUUUGAAGUAUA
siNC	UUCUCCGAACGUGUCACGU
